# Suppressing bias stress degradation in high performance solution processed organic transistors operating in air

**DOI:** 10.1038/s41467-021-22683-2

**Published:** 2021-04-21

**Authors:** Hamna F. Iqbal, Qianxiang Ai, Karl J. Thorley, Hu Chen, Iain McCulloch, Chad Risko, John E. Anthony, Oana D. Jurchescu

**Affiliations:** 1 Department of Physics and Center for Functional Materials, Wake Forest University, Winston Salem, NC USA; 2 Department of Chemistry and Center for Applied Energy Research (CAER), University of Kentucky, Lexington, KY USA; 3 King Abdullah University of Science and Technology, KAUST Solar Center (KSC), Thuwal, Saudi Arabia; 4 Department of Chemistry, Chemistry Research Laboratory, University of Oxford, Oxford, UK

**Keywords:** Electrical and electronic engineering, Electronic devices

## Abstract

Solution processed organic field effect transistors can become ubiquitous in flexible optoelectronics. While progress in material and device design has been astonishing, low environmental and operational stabilities remain longstanding problems obstructing their immediate deployment in real world applications. Here, we introduce a strategy to identify the most probable and severe degradation pathways in organic transistors and then implement a method to eliminate the main sources of instabilities. Real time monitoring of the energetic distribution and transformation of electronic trap states during device operation, in conjunction with simulations, revealed the nature of traps responsible for performance degradation. With this information, we designed the most efficient encapsulation strategy for each device type, which resulted in fabrication of high performance, environmentally and operationally stable small molecule and polymeric transistors with consistent mobility and unparalleled threshold voltage shifts as low as 0.1 V under the application of high bias stress in air.

## Abstract (web summary)

Electrical instability of organic field-effect transistors (OFETs) during operation remains a challenge that limits the device’s real-world technological viability. Here, the authors report a method for diagnosing and suppressing bias stress in solution-processed OFETs operated in air.

## Introduction

Organic semiconductors (OSCs) are emerging as versatile materials that can serve industries ranging from renewable energy to communication, healthcare, security, and space applications. Indeed, molecular and polymeric OSCs will soon become ubiquitous in most sectors of our lives due to their low cost, light weight, and ease of molding into any shape^1–3^. Organic field-effect transistors (OFETs) form the basic building blocks for many of these applications and provide excellent platforms for fundamental studies on charge transport in OSCs^4–8^. Innovations in material and device design have led to OFET performance that is on par with, and even exceeds that, of amorphous silicon transistors, and are sufficient to enable applications like displays, radio frequency identification tags, conformable sensors, health monitoring systems, and memory devices^9–14^. In addition to the performance requirements, the spatial uniformity and stability upon aging, under mechanical, electrical, chemical, and thermal stress, are essential requirements for their commercialization. The uniformity challenge has been tackled through careful processing, and the success has been remarkable^15–17^. OFET stability, however, is a problem that still requires scrutiny^1,18–20^. Electrical instabilities during operation are manifested as a decrease in the drain current, shift in threshold voltage, reduction in charge-carrier mobility, and increase in subthreshold slope, all of which are preventing the deployment of OFETs in technologically relevant applications. The development of environmentally stable materials has led to robust performance when incorporated in devices^21,22^. Addressing the operational stability, however, has proven to be a more difficult task^1,18–20^. Charge trapping has been recognized as the main source of device degradation, a phenomenon that is caused by both external factors (oxidation, moisture, impurities), and intrinsic features (semiconductor molecular structure, structural defects, grain boundaries, voids)^23–25^. The demonstration of bias-stress-free operation in single-crystal devices represents a proof-of-concept that stable OFETs can be achieved in highly ordered OSC layers^26,27^. Subsequent efforts focused on thin-film devices have resulted in remarkable progress through material design, optimization of device architectures, and processing routes^28,29^. For example, Takimiya and collaborators synthesized defect-resilient materials that enabled the fabrication of OFETs with negligible changes in the ON and OFF state currents during 1000 operation cycles^30^. By incorporating molecular additives into the OSC films, Sirringhaus and coworkers displaced the water trapped within the nanometer-sized voids and achieved threshold voltage shifts below 1 V after a day of constant current stress^24^. Kippelen and coworkers fabricated OFETs with gate dielectrics consisting of bilayers of the fluoropolymer Cytop and Al_2_O_3_:HfO_2_ and obtained threshold voltage shifts as low as 0.2 V for over 160 h of operation^31^.

While these achievements mark milestones in OFET research, they are limited to a narrow material space and address only certain device architectures. Engineering stable devices is challenging since charge-carrier trapping leading to instabilities originates from several competing sources and proceeds via complex mechanisms spanning multiple timescales^23^. Practical requirements for OFET stability range from a few minutes for disposable electronics to years for OFET-enabled large-area, flexible displays. Different external factors can accelerate the degradation in application-specific settings, i.e., environment (light, moisture, and temperature), bias or current stress level (current, voltage, and time of continuous operation). Device encapsulation or incorporation of solvent additives during film processing indeed provide good solutions for a subset of materials^24,32,33^, but often fail when transferred to other systems. Clearly, there are no universal strategies that can be adopted, as each system, which consists of a particular material integrated in a specific device structure, is unique and responds differently to external stimuli. Screening for potential degradation pathways is necessary for each case in order to engineer robust devices, but it can be resource and time consuming. Having access to information about the most probable factors and mechanisms triggering device degradation could lead to a fast, rational selection and implementation of the most effective strategy to circumvent the degradation process, and that is unique for each device architecture, composition, and details of operation.

Here, we introduce a method that provides a reliable diagnostic tool for identifying with high accuracy the environmental and operational degradation pathways in several different types of OFETs, and subsequently employ it to eliminate the main sources of instabilities and develop highly stable devices. By monitoring the energetic distribution and time evolution of trap states during OFET operation, and with guidance from density functional theory (DFT) calculations, we gained access to the origins of device degradation, clarified the nature of the traps generated during operation, and the parameter space that gives rise to these traps. This information guided the development of efficient encapsulation pathways that offer selective transparency to some species while blocking the species responsible for degradation. As a result, we delivered robust OFETs with unparalleled operational stability regardless of the composition (small molecule and polymeric OSCs) and configuration (staggered and coplanar), as confirmed by the constant mobility and exceptionally low threshold voltage shift of *ΔV*_th_ = 0.1 eV achieved under aggressive bias stress conditions for 500 min in ambient air.

## Results

### Operational stability measurements on small molecule OFETs

**Fig. 1:**
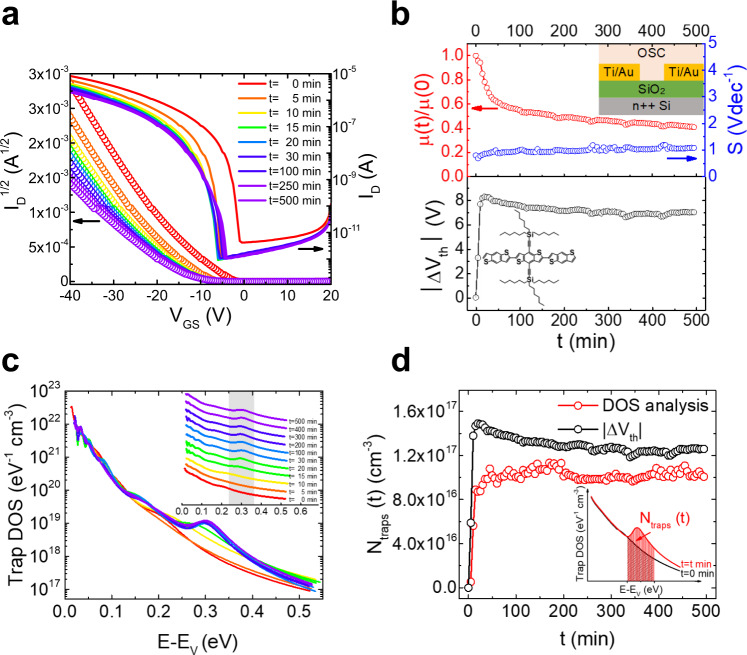
Operational stability tests and real-time trap DOS analysis for TnHS BDT trimer devices performed via repetitive transistor measurements for 500 min in ambient air. **a** Time evolution of the saturation regime transfer characteristics (*I*_D_ vs *V*_GS_ at *V*_DS_ = −60 V). The left and right axes show the square root and the logarithm of *I*_D,_ respectively. The channel length and width of the device are 40 μm and 200 μm, respectively. **b** Time evolution of the mobility *μ* normalized to the value at *t* = 0 min (red), subthreshold slope *S* (blue), and magnitude of threshold voltage shifts |*ΔV*_th_| (black). Insets in the top and bottom panels show the device geometry and the molecular structure of the OSC under study. **c** A subset of the trap DOS spectra evaluated at various times during device operation, offset for clarity in the inset. The curves are plotted as a function of energy from the valence band edge. **d** The generated trap densities during repeated transistor operation evaluated using threshold voltage shifts (black) and the area under the peak in the DOS spectrum (red), as illustrated in the inset.

Operational stability tests were first performed on bottom-contact, bottom-gate OFETs with the semiconducting layer consisting of the small molecule tri(*n*-hexyl)silylethynyl benzodithiophene (TnHS BDT) trimer and SiO_2_ gate dielectric (Fig. 1)^34^. The measurements were performed in ambient air (typical humidity ~40%) and in dark, both by acquiring repeated transfer characteristics (drain current *I*_D_ as a function of gate-source voltage *V*_GS_ at fixed drain-source voltage *V*_DS_)^35,36^, and by recording the transfer curves before and after the application of a prolonged bias stress^22,37,38^. Figure 1a shows the transfer characteristics in the saturation regime of a device during repetitive measurements for 500 min. The red curve corresponds to a fresh device. A drastic change in the slope of the curve and a large negative shift in *V*_th_ is evident within the first 20 min of operation, and then the changes become less prominent. To examine these changes, in Fig. 1b, we plot the evolution of the charge-carrier mobility, *μ*, the subthreshold slope *S*, and the threshold voltage shift, as a function of time. While no statistically relevant changes in the subthreshold slope are observed, a drastic decrease in the mobility (~25%) and large shifts in the threshold voltage (*ΔV*_th_ ~ –8 V) are evident after the first 20 min of operation, subsequently, both parameters remain constant. The reduction in mobility is indicative of charge-carrier trapping in electronic states generated in the bandgap of the OSC^23^. The constant *S* suggests that no significant change occurred in the deep trap distribution, while the shift in *V*_th_ together with the decrease in mobility points to the generation of shallow or intermediate electronic states.

### Trap DOS analysis

In order to access and monitor the time evolution of the energetic distribution of traps, i.e., the trap density of states (DOS) spectrum, we performed the spectral analysis of the density of trap states^23^. Figure 1c shows the trap DOS spectra plotted as a function of energy with respect to the valence band edge for a device measured in air. Each curve was evaluated at different times during transistor operation (a subset of the spectra is shown here for clarity and a complete set, as well as the functions used in the analysis, are provided in Supplementary Fig. 1). At *t* = 0 min (red curve) and *t* = 5 min (orange curve), the DOS resembles an exponential function, with the majority of electronic states distributed in the vicinity of the band edge. This type of trap distribution is typical for polycrystalline OSCs and the electronic states tailing into the bandgap arise from disorder within the crystal due to thermal molecular motions and/or structural defects^23^. At *t* = 15 min (yellow curve), the formation of a peak at 0.31 eV is evident, which is then followed by an increase in the peak amplitude starting at *t* = 20 min (green curve). This peak is the result of the generation of discrete electronic states in the bandgap of the OSC during operation and its location (i.e., several *kT* from the band edge, where *k* is the Boltzmann constant and *T* is the temperature) suggests that the traps that forms lie at intermediate energies, between shallow and deep. The DOS spectra remain unchanged after 20 min, indicating that no additional electronic states are generated during the remaining period of transistor operation, which explains why the device parameters remain constant. We modeled the DOS curves in Fig. 1c using two exponential functions to describe the distribution of shallow and deep states, respectively, with the addition of a Gaussian distribution to account for the presence of the peak. A 50% increase in the shallow trap density between the first and last measurement, and an order of magnitude increase in the deep trap density, respectively, is evident, along with a 30% and 24% decrease in the characteristic width of the shallow and deep states, respectively (Supplementary Table 1, Supplementary Fig. 2). This narrowing of the DOS occurs to accommodate the additional electronic states generated during operation that causes the peak in the DOS spectrum that is accounted for by the Gaussian centered at 0.31 eV. To quantify the increase in the density of traps as a function of time (*N*_trap_ *(t)*), and hence determine the number of traps generated during operation, and that are responsible for the device degradation, the difference between the area under the peak of the DOS spectrum and the baseline spectrum (at *t* = 0 min) was evaluated for each curve; the results are shown in Fig. 1d, along with the value of the generated trap densities determined from the shifts in *V*_th_. After a subsequent rest period, the mobility and the threshold voltage were recovered (Supplementary Fig. 3a) and the trap DOS spectrum became identical to that of the fresh device (Supplementary Fig. 3b), suggesting that the generated electronic states are metastable and persist only during operation.

**Fig. 2:**
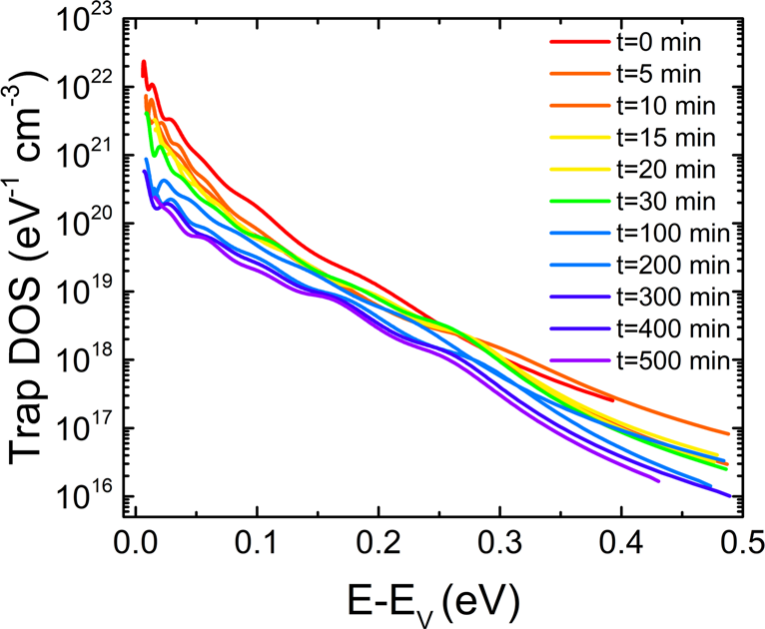
Time evolution of the DOS spectra during 500 min of transistor operation in vacuum for a TnHS BDT trimer device. A subset of the trap DOS spectra evaluated at various times during operation are shown. The curves are plotted as a function of energy from the valence band edge.

The above experimental observations and analysis clarified that the source of device instabilities is charge trapping in intermediate bandgap states occurring under operation in air. To establish the precise nature of the predominant traps and the factors that lead to their formation, we performed several experiments and computational analyses. First, we repeated the measurements under vacuum (∼10^−6^ Torr) to decouple the environmental and operational degradation processes and investigate the sole effect of bias stress on the device performance and trap dynamics. The evolution of the DOS spectrum is included in Fig. 2. Clearly, a peak does not form, confirming that bias stress alone is not responsible for the generation of electronic traps around 0.31 eV observed for the device tested in air. A 50% decrease in the density of deep traps with a narrowing of the DOS by ~10%, and a two orders of magnitude reduction in shallow trap densities, with a 50% decrease in the characteristic width, occur after 500 min of operation (Supplementary Table 2 and Supplementary Fig. 4). The reduction in the trap density is a consequence of the de-gassing step that removes air from the film. The likely candidate for doping is residual oxygen (O_2_), which can cause p-type doping as observed in many small molecules and conjugated polymers^39^. Oxygen doping is also supported by the changes in the current–voltage characteristics of the device in ambient compared to vacuum (Supplementary Fig. 5), i.e. the increase in OFF current by two orders of magnitude and a large positive shift in the threshold voltage (*ΔV*_th_ = 26 V). In Supplementary Fig. 6 we include a comparison of the evolution of device properties during operational stability tests performed under vacuum, and ambient, respectively. While the mobilities decay at similar rates, larger shifts in *V*_th_ are observed in vacuum. P-dopants create acceptor-like states in the bandgap, increasing the hole concentration in the transistor channel and in their absence, a higher gate-voltage is required to accumulate mobile charges. With time, the shifts in *V*_th_ become larger as more dopant molecules are gradually evacuated, until all dopants are removed, and the shifts saturate. This observation highlights that the presence of O_2_ molecules, i.e., oxygen doping, is necessary in generating the required charge concentration to yield high performance in these devices.

We then examined the effect of environmental contaminants on the DOS spectrum and device stability by eliminating bias stress. Freshly made devices were measured in air, then stored under ambient air, in dark, and the transfer characteristics were recorded daily over a period of 40 days. The device parameters remain constant (Supplementary Fig. 7a), the DOS spectra did not show any statistically relevant change, and the peak observed under continuous operation in ambient is absent (Supplementary Fig. 7b). These cumulative measurements suggest that the electronic states leading to device degradation are generated only when bias stress and environmental effects are simultaneously present and are not caused by intrinsic structural and energetic disorder in the OSC. This is an important result because it confirms that the OSC itself can have a good shelf-life in the absence of bias stress, and thus can yield stable devices provided the device degradation mechanisms are clearly identified and eliminated.

### DFT calculations to predict defect formation

The questions that arise are the following: (1) What is the nature of the environmental species and the process by which they cause electronic traps that lead to device degradation under operation? (2) How can this process be inhibited through device design in order to achieve stable devices? Before answering these questions, it is informative to review the studies on operational instabilities. Chemical reactions between the OSC and chemical species present in ambient air can generate trap states and the effect is more severe in the presence of bias stress^40,41^. Tsetseris et al. investigated the interaction of O_2_ and H_2_O with pentacene crystals using first-principle calculations and found that O_2_ dissociation and formation of oxygen complexes generate bandgap states at 0.33–0.40 eV above the valence band edge, while H_2_O remains intact between molecular layers in the crystal^42^. Lang et al. experimentally detected bias-induced hole trap states at 0.38 eV using space charge limited current measurements^43^. Knipp and Northrup reported on gap states in pentacene at 0.29 eV as a result of an electrochemical process facilitated by the presence of O_2_ and the applied field^40^. In the presence of water, several mechanisms for electrical instabilities have been postulated. For example, polaron formation in the outermost layer due to the polarization of surrounding water molecules results in the stabilization of holes leading to the creation of polaron traps states^44^. In OFETs with SiO_2_ dielectric, charge trapping due to absorbed water increases the severity of the bias stress degradation, and surface passivation with self-assembled monolayers has been adopted to reduce this effect^20,37,45^. In the case of the donor–acceptor copolymer indacenodithiophene-co-benzothiadiazole (IDT-BT), the interaction of water molecules with the polymer backbone broadens the distribution of torsion angle of the bond between the IDT and BT sub units and increases the energetic disorder by over 200 meV, creating shallow trap states for holes^24^. Weitz and coworkers showed that the self-ionization of liquid water under bias stress creates a dominant trap that causes threshold shifts in OFETs based on diketopyrrolopyrrole^46^. Another mechanism for threshold voltage instabilities in the presence of water is proton migration^47^.

**Fig. 3:**
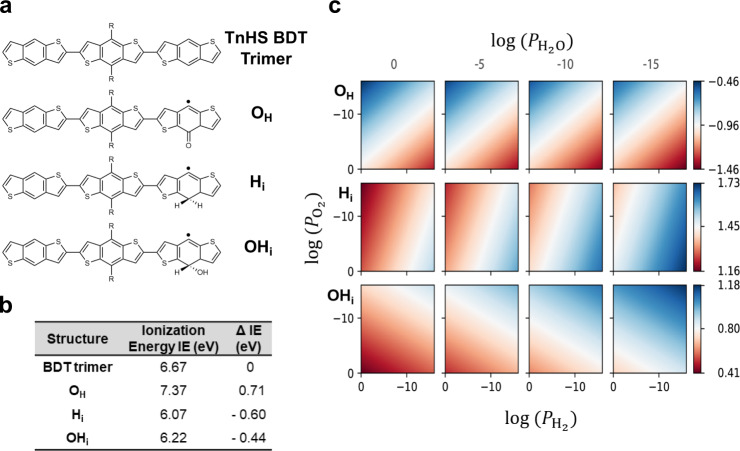
Chemical structures of the TnHS BDT trimer and potential chemical defects, and their corresponding ionization energies and defect formation energies. **a** Chemical structures of the neutral molecule and potential chemical defects via addition reactions of the TnHS BDT trimer. **b** Ionization energies of the TnHS BDT trimer and the respective chemical defect structures, **c** Chemical defect formation energy (in eV) as a function of ${P}_{{{\rm{O}}}_{2}},{P}_{{{\rm{H}}}_{2}},{P}_{{{\rm{H}}}_{2}{\rm{O}}}$ in Pa for, from top to bottom, O_H_, H_i_, and OH_i_.

While we do not rule out the above-proposed mechanisms, we evaluated three chemical defects that are hypothesized as being the most probable to form and hence may be responsible for the trap states in the TnHS BDT trimer, as shown in Fig. 3a. The radical formed by oxygen substitution is denoted as O_H_, and those with hydrogen/hydroxyl addition are represented by H_i_ and OH_i_, respectively, and the neutral molecule is also shown as reference. Molecular DFT calculations indicate that both H_i_ and OH_i_ defect structures have lower (adiabatic) ionization energies with respect to TnHS BDT trimer (see table in Fig. 3b), while a significant positive ionization potential shift (~ 0.71 eV) is found for the O_H_ defect structure, which makes it less likely to be responsible for trap states above the valence band^23^. To evaluate the relative thermodynamic stabilities of these defects, their formation energies (${E}^{{\rm{f}}}$) were calculated as a function of oxygen, hydrogen, and water partial pressures; the results are shown in Fig. 3c. We note that we spanned a wide range of partial pressures necessary due to the difficulty in providing reliable estimates for gas compositions in organic solids, especially considering the relatively large “empty” space in TnHS BDT trimer crystal (~71.6% space fill based on van der Waals molecular volume). Further, to avoid the assumption of thermodynamic equilibrium in organic solids between hydrogen, oxygen, and water, the normalization scheme for the chemical potential calculation from Herrmann et al. was adopted^48^. The formation energy of the O_H_ defect, ${E}^{{\rm{f}}}\left({\mathrm{{O}}}_{{\mathrm{H}}}\right)$, remains negative for all (${P}_{{{\rm{O}}}_{2}},{P}_{{{\rm{H}}}_{2}},{P}_{{{\rm{H}}}_{2}{\rm{O}}}$) considered, corroborating the argument that O_H_ defects are not responsible for the traps observed, otherwise the peak in DOS spectra would have appeared even in the absence of bias stress. In general, for neutral defects, the formation energies follow ${E}^{{\rm{f}}}\left({{\rm{O}}}_{{\rm{H}}}\right) < {E}^{{\rm{f}}}\left({{\rm{H}}}_{{\rm{i}}}\right) < {E}^{{\rm{f}}}\left({{{\rm{OH}}}}_{{\rm{i}}}\right)$. ${E}^{f}$ is linearly dependent on ${{\log }}\left(P\right)$ (Supplementary Fig. 8, SI), which indicates that ${E}^{f}$ for H_i_ and OH_i_ defects can be raised simultaneously by lowering the water composition of the environment. The thermodynamic ($0/+$) transition level is calculated to be 0.341 eV and 0.136 eV above the valence band edge for the charged H_i_ and OH_i_ defects, respectively (Supplementary Fig. 9a), which suggests H_i_ defects are more likely to be responsible for the observed hole traps. The ($0/-$) transition levels reside at 0.94 eV and 0.72 eV above the valence band edge for H_i_ and OH_i_, respectively. Such levels are too deep in the bandgap to act as effective donors.

### Encapsulant selection for trap elimination

**Fig. 4:**
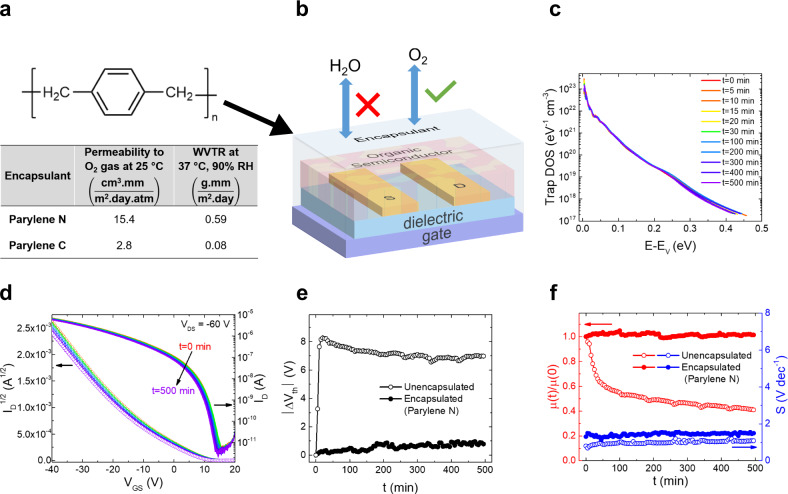
Device encapsulation and operational stability tests on encapsulated small-molecule OSC bottom-contact, bottom-gate devices in ambient air. **a** Molecular structure of parylene N (top) and barrier properties of different types of parylene (bottom)^49^. **b** Schematic of a device encapsulated with parylene N, which selectively allows some O_2_ molecules to pass through, while effectively preventing the permeation of H_2_O molecules. **c** Trap DOS spectrum of an encapsulated device evaluated at various times during repeated operation in ambient air. **d** Time evolution of the magnitude of threshold voltage shifts |*ΔV*_th_| of an encapsulated device (solid circles) in comparison to an unencapsulated device (open circles). **e** Time evolution of the mobility *μ* normalized to the value at t = 0 min (red), and subthreshold slope *S* (blue), of an encapsulated device (solid circles) in comparison to an unencapsulated device (open circles). **f** Time evolution of the saturation regime transfer characteristics (*I*_D_ vs *V*_GS_ at *V*_DS_ = −60 V) during repeated transistor operation. The left and right axes show the square root and the logarithm of *I*_D,_ respectively.

Having identified the nature and microscopic origins of the trap states pertaining to device degradation, we used the insights gained from this study to implement a strategy to enhance the operational stability of the device. Since moisture generated the electronic traps and is thus, the culprit for device degradation, but oxygen is needed to enhance the performance through doping, a device encapsulation strategy that shields water molecules from diffusing into the OSC, but is permeable to oxygen, is necessary. One compound that facilitates these requirements is the polymer poly-*para*-xylene, known as parylene (molecular structure in Fig. 4a)^49^. Parylene can form conformal, pinhole-free coatings and can be deposited by vapor deposition onto surfaces maintained at room temperature, and thus, it is not restricted in terms of the nature of the OSC or substrate. Several types of parylene derivatives exist; the table in Fig. 4a summarizes the permeability to O_2_ as well as the water vapor transmission rate (WVTR) of parylene N and parylene C. Following the insights gained from our DOS and DFT analyses, we chose parylene N as an encapsulant layer for our device because of its higher permeability to O_2_ gas (Fig. 4b). The DOS spectra of the encapsulated devices showed no evidence of a peak during the entire 500 min of operation, and the curves coincide remarkably well, confirming the fact that no change in the trap states occurs (Fig. 4c). The stability of the device can also be witnessed in the transfer characteristics acquired during prolonged transistor operation (Fig. 4d). The time evolution of the threshold voltage shift shown in Fig. 4e depicts a shift of less than 1 V (${\triangle V}_{{\rm{th}}} \sim 0.9$ V), a value significantly lower than that measured in an unencapsulated device (${\triangle V}_{{\rm{th}}} \sim 8.0$ V), or when the less efficient encapsulant parylene C was used (${\triangle V}_{{\rm{th}}} \sim 2.5$ V) (see Supplementary Fig. 10). The other device parameters were very stable as well, with a constant mobility and subthreshold slope being recorded during 500 min of continuous operation in ambient conditions for the OFETs encapsulated in parylene N (see Fig. 4f). Supplementary Fig. 11 shows the hysteresis in the current–voltage characteristics of an encapsulated device in comparison to an unencapsulated device, with the former exhibiting negligible hysteresis in both the transfer and output characteristics.

### Bias stress measurements on optimized devices

**Fig. 5:**
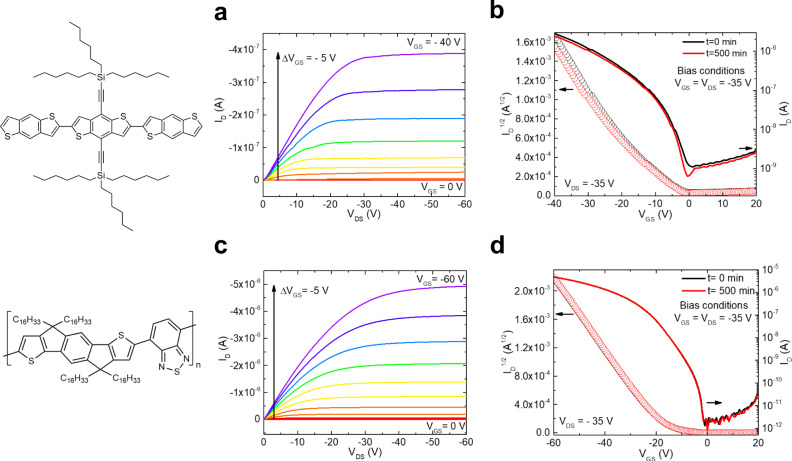
Bias stress measurements on encapsulated small molecule and polymer OFET devices. **a** Output characteristics of a small molecule OSC device at various fixed gate-voltages. The molecular structure of the molecule, namely TnHS BDT trimer, is also included. **b** Transfer characteristics of the same device acquired at *V*_DS_ = −35 V prior to stressing (black) and after 500 min of stressing (red) under the application of a continuous drain voltage and a dynamic gate bias pulsed at 10 s interval at *V*_DS_ = *V*_GS_ = −35 V. **c** Output characteristics of a polymer device at various fixed gate-voltage. The molecular structure of the polymer, namely IDT-BT, is also included. **d** Transfer characteristics of the same IDT-BT device acquired at *V*_DS_ = −35 V prior to stressing (black) and after 500 min of stressing (red) under the application of a continuous drain voltage and a dynamic gate bias pulsed at 10 s interval at *V*_DS_ = *V*_GS_ = −35 V.

The stability studies reported thus far were performed via repeated transfer scans, with the main objective of elucidating the causes and mechanism of device degradation. Many applications based on OFETs, however, require constant biasing, with the details depending on the specific circuit that they serve. For example, active matrix OLED displays are current-driven and require the application of a constant current stress^1,24^. On the other hand, voltage-driven display technologies such as liquid crystal or electrophoretic displays require the devices to operate in a pulsed mode with bias voltages applied alternatingly between ON and OFF states^1,31^. To assess the reliability of our devices in an application-specific setting, we further performed operational stability tests by replicating the biasing conditions in voltage-driven display technologies. Figure 5a shows the output characteristics of a device encapsulated in Parylene N prior to electrical stressing. A continuous drain bias and a dynamic gate bias pulsed at 10 s interval was applied for a period of 500 min under high bias conditions in the saturation regime (*V*_GS_ = *V*_DS_ = −35 V). The transfer characteristics confirm the excellent operational stability of the device under these biasing conditions, with a threshold voltage shift of *ΔV*_th_ = −0.2 V and a decrease in the mobility of less than 6%, while the other parameters remain unchanged (Fig. 5b). The transfer characteristics of a device encapsulated with parylene C and stressed under identical bias conditions are displayed in Supplementary Fig. 12a in SI. The threshold voltage stability of this device (i.e., *ΔV*_th_ = −1.3 V) is superior to an unencapsulated device (Supplementary Fig. 12b, *ΔV*_th_ = +4.9 V) but is greatly inferior to the device encapsulated with paralyne N (Fig. 5b, *ΔV*_th_ = −0.2 V) due to the lower O_2_ permeability characteristic to parylene C compared to parylene N (see Table in Fig. 4a). Since we found that traces of O_2_ are necessary for TnHS BDT to operate efficiently, these results highlight the importance of a holistic choice of encapsulant for achieving stable devices.

### Expanding the method to polymer OFETs

**Fig. 6:**
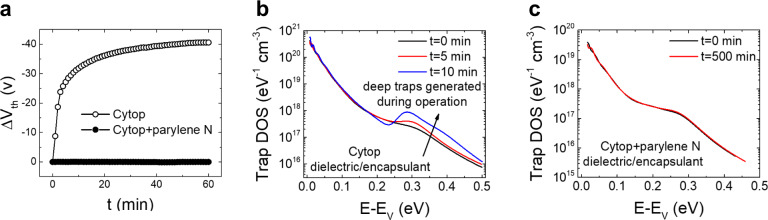
Comparison of threshold voltage shifts and trap DOS in IDT-BT devices fabricated with different dielectrics, which also play the role of encapsulants, during repeated transistor operation for 500 min in ambient air. **a** Time evolution of the shift in threshold voltage of a Cytop-only device (open circles) compared to the bilayer Cytop/parylene dielectric device (solid circles). **b** Trap DOS spectra of a Cytop-only device evaluated at different times during transistor operation shows the generation of discrete bandgap states at ca. 0.28 eV after 10 min of transistor operation. **c** Trap DOS spectra of a bilayer dielectric device evaluated at different times during transistor operation indicates no change in the DOS.

We further explored the universality of our strategy by expanding our study to polymer semiconductor OFETs. We focused on IDT-BT, which, in the presence of environmental species behaves similarly to the TnHS BDT trimer^24^. IDT-BT OFETs are typically fabricated in the bottom-contact, top-gate architecture with Cytop dielectric (Supplementary Fig. 13a, SI)^24,50^, which is also the geometry adopted here. While Cytop makes a good interface with IDT-BT copolymer in OFETs, yielding charge carriers mobilities in excess of 1 cm^2^ V^−1^ s^−1 50,51^, its encapsulation properties are inferior to that of parylene^52^, making the devices susceptible to environmental contaminants and thereby unstable^24^. In order to maintain the good performance facilitated by the Cytop dielectric and simultaneously prevent degradation, we fabricated devices with a double layer dielectric consisting of a thin film of parylene evaporated over Cytop (Supplementary Fig. 13b). The output characteristics are shown in Fig. 5c. The device stressed for 500 min in ambient air under *V*_GS_ = *V*_DS_ = −35 V shows very little change in the transfer characteristics (Fig. 5d); the mobility and subthreshold slope remained constant at 2.2 cm^2^ V^−1^ s^−1^ and 1.2 V dec^−1^, respectively, and an exceptionally small shift is recorded in the threshold voltage (*ΔV*_th_ = +0.1 V). In addition, this device shows very low hysteresis in both transfer and output characteristics (Supplementary Fig. 14 in SI). The threshold voltage under repeated measurements was also stable, with shifts remaining below 0.1 V (Fig. 6a), as opposed to the Cytop-only devices, where shifts as large as −40 V were recorded. The subthreshold slope and mobility also remained constant upon repetitive measurements, at ~4.0 cm^2^ V^−1^ s^−1^ and ~1 V dec^−1^, respectively (Supplementary Fig. 15a, SI), with the mobility values being independent of the applied gate-voltage (Supplementary Fig. 15b). In devices with only Cytop as dielectric/encapsulant, the trap DOS spectra indicate that discrete bandgap states are gradually being generated, as confirmed by the overall increase in DOS and the formation of a peak at ca. 0.28 eV from the valence band edge during operation (Fig. 6b). On the other hand, the use of Cytop/Parylene N bilayer dielectric/encapsulant yield devices with a lower trap density that remains constant under operation and hence exhibit remarkable operational stability (Fig. 6c).

## Discussion

By monitoring the trap DOS in real time and with guidance from DFT calculations, we have identified the processes causing OFET environmental and operational device degradation. These insights allowed us to rationally design solutions for eliminating/reducing the degradation and consistently achieve stable OFETs regardless of the device structure and the nature of the OSC. Device encapsulation layers were selectively chosen for each system to provide a barrier for the most probable and detrimental source of electronic traps, which completely suppressed the formation of trap states in the OSC bandgap, and yielded stable devices with near-ideal current–voltage characteristics, constant mobilities and unprecedented threshold voltage shifts of less than 0.1 V achieved under the application of high bias stress for 500 min in ambient air. This study provides unparalleled insights into the physical processes taking place during prolonged device operation and the results are consistent for both small molecule and polymeric solution-processed OSCs incorporated in devices with a top or bottom-gate geometry. Our results demonstrate a pathway to overcome one of the grand challenges in organic electronics and enable the establishment of practical guidelines for the design of materials and device architectures that yield stable, high-performance OFETs.

## Methods

### Sample and device fabrication

Bottom-gate, bottom-contact OFETs were fabricated on a heavily doped n-type Si wafer with a 200 nm layer of thermally grown SiO_2_ serving as the gate and the gate dielectric, respectively. A 3 nm adhesion layer of Ti followed by 40 nm of Au were then deposited via e-beam and thermal evaporation, respectively, through a shadow mask to define the source and the drain electrodes. Prior to depositing the OSC layer, the substrates with predefined contacts were cleaned thoroughly by immersing in a hot acetone bath followed by a hot isopropanol bath, both maintained at 85 °C for 10 min, and then subjected to a UV-ozone treatment for 10 min. The substrates were rinsed with deionized water and dried under N_2_ gas to remove any remaining residue after the UV-ozone treatment. A self-assembled monolayer, pentafluorobenzenethiol (PFBT, Millipore Sigma) was applied to the contacts by immersing the cleaned test pads in a 30 mM solution of PFBT in ethanol for 30 min. After the treatment, the test pads were thoroughly rinsed with ethanol and dried with a stream of N_2_ gas. TnHS BDT trimer films were spin coated from a 1.5 wt % solution in chlorobenzene at 1000 r.p.m for 80 s. The completed device was placed in a vacuum desiccator for 2 h to remove any residual solvents. Encapsulated TnHS BDT trimer devices were made by evaporating a layer of parylene N or parylene C (~1500 nm) over the device. For top-gate, bottom-contact OFETs, the source and drain contacts were deposited following the same procedures. Then, IDT-BT films were spin coated from a 5 mg/mL solution in chlorobenzene at 2000 r.p.m for 60 s. The resulting films were annealed on a hot plate at 100 °C for 15 min to remove residual solvents. To complete the top-gate devices, a 940 nm layer of Cytop from AGC Inc. (CTL-809-M) was spin coated over the IDT-BT films at 2000 r.p.m. for 60 s. The films were then annealed on a hot plate at 100 °C for 20 min and placed in a vacuum desiccator overnight at room temperature. A 50 nm layer of Au was then thermally evaporated through a shadow mask to form the top gate. The bilayer dielectric devices were fabricated by evaporating a 650 nm layer of parylene N over Cytop prior to the top-gate electrode evaporation.

Parylene N or parylene C evaporation was carried out in a bespoke reactor consisting of an evacuated quartz tube (pressure ~1 mTorr) maintained at three different temperature zones. First, the dimer di-*para*-xylene (Acros Organics) was heated at 120 °C to sublime into a dimeric gas which passed through a hot furnace maintained at 700 °C. The dimer then dissociated into its gaseous monomers inside the furnace. Finally, the monomeric gases polymerized onto the device placed in the third region of the tube maintained at room temperature forming a uniform conformal coating.

### Electrical characterization

OFETs were electrically characterized using an Agilent 4155C Semiconductor Parameter Analyzer. All measurements were performed in dark, at room temperature and in ambient air, except for the measurements under vacuum, which were performed in a vacuum probe station. The charge-carrier mobility was determined from the slope of the √*I*_D_ vs. *V*_GS_ plot using the following equation^7^,

1 $${\rm{\mu }}=\frac{2L}{W{C}_{{\rm{i}}}}{\left(\frac{\partial \sqrt{{I}_{{\rm{D}}}}}{\partial {V}_{{\rm{GS}}}}\right)}^{2}$$

where *L* and *W* are the length and width of the transistor channel. The threshold voltage was evaluated by extrapolating the linear fit of √*I*_D_ vs *V*_GS_ to *I*_D_ = 0. Only devices with ideal or near-ideal current–voltage characteristics have been considered in the analysis, which was the majority of our devices.

### Stability tests

Operational stability measurements in ambient air were performed in dark and at room temperature. The relative humidity varied from 32 to 35%. Operational stability tests in vacuum were performed by placing the device in a vacuum probe station which was pumped down for 24 h to a pressure of 10^−6^ Torr prior to acquiring measurements. The tests were performed using two methods. In the first method, the saturation regime transfer characteristics (*I*_D_ vs. *V*_GS_ at *V*_DS_ = −60 V) were recorded repeatedly for a period of 500 min. The gate-source voltage was swept from 20 V to −40/−60 V in incremental steps of −0.5 V. In the second method, a continuous drain bias and a dynamic gate bias pulsed at 10 s interval were applied to the device for a period of 500 min at *V*_GS_ = *V*_DS_ = −35 V and the saturation regime transfer characteristics were acquired both before and after the stressing. The tests were performed on over 15 devices of each sample type and the results were consistent.

For the environmental stability tests, the device was stored in a container covered with aluminum foil in order to shield light. The sample was placed in ambient for a period of 40 days and each day the transfer characteristics were acquired in ambient air and in dark.

### Trap DOS analysis

The underlying principle behind the extraction of the trap DOS as a function of energy in the bandgap of the OSC is the gate-voltage-dependent energy level bending occurring at the OSC/dielectric interface. This energy level bending induces a gate-voltage-dependent potential difference called the interface potential *V*_0_(*U*_GS_). The linear regime transfer characteristics were recorded, from which the gate-voltage-dependent channel conductivity was evaluated using,

2 $${\rm{\sigma }}\left({U}_{{\rm{GS}}}\right)=\frac{L}{W}\frac{{I}_{{\rm{D}}}}{{V}_{{\rm{DS}}}}$$

Here *U*_GS_ is the gate-source voltage above the flat-band voltage *V*_FB_. i.e., *U*_GS_ = |*V*_GS_−*V*_FB_|. *V*_FB_ is assumed to be the turn-on voltage (*V*_ON_) of the device. The interface potential function *V*_0_ *(U*_GS_*)* was then obtained by numerically solving the following equation (see ref. ^53^. for a complete derivation):

3 $${{\exp }}\left(\frac{e{V}_{0}}{{kT}}\right)-\frac{e{V}_{0}}{{kT}}-1=\frac{e}{{kT}}\frac{{{\rm{\varepsilon }}}_{{\rm{i}}}d}{{{\rm{\varepsilon }}}_{{\rm{s}}}l{{\rm{\sigma }}}_{0}}[{U}_{{\rm{GS}}}{\rm{\sigma }}\left({U}_{{\rm{GS}}}\right)-\int_{0}^{{U}_{\rm{GS}}}\sigma \left(\widetilde{{U}_{{\rm{GS}}}}\right)d\widetilde{{U}_{{\rm{GS}}}}]$$

where *ε*_*i*_ and *l* are the relative permittivity and the thickness of the dielectric, respectively, *ε*_*s*_ and *d* are the relative permittivity and the thickness of the semiconductor, respectively and *σ*_*0*_ is the conductivity at flat band voltage. The function *V*_0_ *(U*_GS_*)* was used to determine the total hole density from,

4 $$p\left({V}_{0}\right)=\frac{{\varepsilon }_{0}{\varepsilon }_{i}^{2}}{{\varepsilon }_{s}{l}^{2}e}{U}_{{\rm{GS}}}{\left(\frac{d{V}_{0}}{d{U}_{{\rm{GS}}}}\right)}^{-1}$$

The trap DOS was then obtained by numerically differentiating the total hole density with respect to *V*_*0*_. i.e.,

5 $$N(E){\rm{\approx }}\frac{1}{e}\frac{{dp}({V}_{0})}{d{V}_{0}}$$

where *E* *=* *eV*_0_ is the energy of the trapping state with respect to the Fermi level. The trap DOS was then plotted as a function of energy from the valence band edge, i.e., *E*−*E*_V_ = (*E*_V_−*E*_F_)−(*E*−*E*_F_) The assumption that at maximum *U*_GS_, the quasi-Fermi level coincides with the valence band edge allowed the estimation of *E*_V_−*E*_F_ to be 0.64 eV. We note that the above assumption introduces uncertainty in the trap depth. However, since our hypotheses are based not on the absolute DOS but on the changes in the DOS of a device under external stimuli (electrical stress and environmental contaminants), the uncertainty in the trap depth does not impact our conclusions.

The density of traps generated during transistor operation was calculated from the threshold voltage shift using,

6 $${N}_{{\rm{trap}}}\left(t\right)\approx \frac{{C}_{i}\triangle {V}_{{\rm{th}}}(t)}{e}$$

where *C*_*i*_ is the areal capacitance of the dielectric (17.3 nF for 200 nm of SiO_2_) and *e* is the electronic charge. The volume density of trap states (in units of cm^−3^) was evaluated using the thickness of the TnHS BDT trimer films (58.6 nm).

The experimental DOS curves were modeled using two exponential functions to describe the distribution of shallow and deep states, respectively, with the addition of a Gaussian distribution to account for the presence of the peak, according to the following equation:

7 $$N\left(E\right)={N}_{1}{{\exp }}\left(-\frac{E}{{E}_{1}}\right)+{N}_{2}{{\exp }}\left(-\frac{E}{{E}_{2}}\right)+A\,{{\exp }}\left[-\frac{{\left(E-{E}_{{\rm{peak}}}\right)}^{2}}{2{\sigma }^{2}}\right]$$

where *E*_1_, *E*_2_ are the characteristic decay energies, *N*_1_, *N*_2_ are the amplitudes of the respective exponential distributions, *E*_peak_ defines the position of the Gaussian distribution with an amplitude *A* and standard deviation *σ*.

### Defect formation energy and transition level

The formation energy of a defect D with charge q,${E}^{{\rm{f}}}\left(D,q\right)$, is defined as:

8 $${E}^{f}\left(D,q\right)={E}_{{\rm{tot}}}\left(D,q\right)-{E}_{{\rm{tot}}}\left(P\right)-{\Sigma }_{i}{n}_{i}{{\rm{\mu }}}_{i}+q{E}_{{\rm{F}}}+{E}_{\text{corr}}$$

where ${E}_{{tot}}\left(P\right)$ and ${E}_{{tot}}\left(D,q\right)$ are the total energy of pure TnHS BDT trimer bulk structure and that with defect *D*, respectively. Total energies were obtained via DFT calculations as implemented in Gaussian 16^54^, and Vienna *Ab-initio* Simulation Package^55^ (Supplementary Discussion 4). ${E}_{{\rm{F}}}$ is the Fermi energy, ${n}_{i}$ is the stoichiometry coefficient of species *i* in the defect formation reaction, and ${\mu }_{i}$ is its chemical potential. ${E}_{\text{corr}}$ is a correction term due to the finite size effect in charged defect calculations. While this term can be accurately evaluated for localized charged state, in Supplementary Fig. 9a the correction term is set to zero as in the case of TnHS BDT trimer charged defects are not well-localized at an atomic site, and the dielectric tensor is not isotropic. We note that the correction term calculated from a localized charge model should not alter the argument qualitatively, as shown in Supplementary Fig. 9b (see its caption for detailed implementations), where the ($+/0$) transition level of H_i_ is still closer to the experimentally observed trap DOS peak than that of OH_i_. All thermodynamic quantities are calculated at 298.15 K.

For neutral defects, ${q}=0$, thus, ${E}^{{\rm{f}}}$ is not dependent on ${E}_{{\rm{F}}}$. As the equilibrium between environmental gases cannot be assumed, chemical potentials of oxygen, hydrogen and water enter the ${\Sigma }_{{\rm{i}}}{n}_{{\rm{i}}}{\mu }_{{\rm{i}}}$ term independently, as shown in Eq. 9.

9 $${\Sigma }{\varSigma }_{i}{n}_{i}{\mu }_{i}={n}_{{{\rm{H}}}_{2}}{\mu }_{{{\rm{H}}}_{2}}+{n}_{{{\rm{O}}}_{2}}{\mu }_{{{\rm{O}}}_{2}}+{n}_{{{\rm{H}}}_{2}{\rm{O}}}{\mu }_{{{\rm{H}}}_{2}{\rm{O}}}$$

The complication arising from Eq. 9 is there are more environmental species than stoichiometric relations, i.e., there are infinite choices of $\left({n}_{{{\rm{H}}}_{2}},{n}_{{{\rm{O}}}_{2}},{n}_{{{\rm{H}}}_{2}{\rm{O}}}\right)$ such that the defect formation reaction is balanced, leading to arbitrary dependence on ${{\rm{\mu }}}_{{H}_{2}}$ for ${E}^{{\rm{f}}}\left(D,q\right)$. This problem is solved by using the normalization scheme proposed by Herrmann et al. which adds another constraint such that the species counts in defect formation reaction is orthogonal to that in water formation reaction, yielding unique choice for $\left({n}_{{{\rm{H}}}_{2}},{n}_{{{\rm{O}}}_{2}},{n}_{{{\rm{H}}}_{2}{\rm{O}}}\right)$^48^. For charged defects, ${E}^{{\rm{f}}}\left(D,q\right)$ becomes a function of ${E}_{{\rm{F}}}$, and their relative stability at a certain ${E}_{{\rm{F}}}$ can be evaluated based on the thermodynamic transition level $\left(q/{q}^{{\prime} }\right)$:

10 $$\left(q/{q}^{{\prime} }\right)=\frac{{E}^{{\rm{f}}}\left(q,{E}_{{\rm{F}}}\right)-{E}^{{\rm{f}}}\left(q,{E}_{{\rm{F}}}\right)}{{q}^{{\prime} }-q}$$

which is the Fermi-level position at which the defect formation energies of charge $q$ and ${q}^{{\prime} }$ are equal.

## Supplementary information

Supplementary Information

## Data Availability

All data supporting the conclusions in the paper are present in the paper and/or in Supplementary Information. The data sets generated during and/or analyzed during the current study are available from the corresponding author upon reasonable requests.
