# Supplementary material for: Suppressing bias stress degradation in high performance solution processed organic transistors operating in air
Source: Nat Commun. 2021 Apr 21;12:2352. doi: 10.1038/s41467-021-22683-2 (PMC8060299; doi:10.1038/s41467-021-22683-2)
Supplement: Supplementary file 1 — Supplementary Information [file 41467_2021_22683_MOESM1_ESM.pdf]

# Supplementary Information

# Suppressing bias stress degradation in high performance solution processed organic transistors operating in air

Hamna F. Iqbal[1], Qianxiang Ai[2], Karl J. Thorley[2], Hu Chen[3], Iain McCulloch[3,4], Chad Risko[2], John E. Anthony[2] and Oana D. Jurchescu[1,*]

[1]Department of Physics and Center for Functional Materials, Wake Forest University, Winston Salem, NC 27109, USA

[2]Department of Chemistry and Center for Applied Energy Research (CAER), University of Kentucky, Lexington, KY 40506, USA

[3]King Abdullah University of Science and Technology, KAUST Solar Center (KSC), Thuwal 23955-6900, Saudi Arabia

[4]Department of Chemistry, Chemistry Research Laboratory, University of Oxford, Oxford, OX1 3TA, UK

*Corresponding author email: Jurchescu@wfu.edu

**Contents:**

Supplementary Discussion 1: Operational stability tests on unencapsulated TnHS BDT trimer devices in ambient air……………………………………………………………………...2

Supplementary Discussion 2: Operational stability tests on unencapsulated TnHS BDT trimer devices in vacuum…………………………………………………………………………4

Supplementary Discussion 3: Environmental stability tests on unencapsulated TnHS BDT trimer devices……………………………………………………………………………………..6

Supplementary Discussion 4: Computational details…………………………………………...7

Supplementary Discussion 5: Operational stability tests on unencapsulated vs. encapsulated TnHS BDT trimer devices in air……………………………………………………………………….9

Supplementary Discussion 5: Hysteresis in current-voltage characteristics of TnHS BDT trimer devices………………………………………………………………………………………10

Supplementary Discussion 6: Bias stress measurements on TnHS BDT trimer devices…………11

Supplementary Discussion 7: Bilayer dielectric IDT-BT devices………………………………..12

## Supplementary Discussion 1: Operational stability tests on unencapsulated TnHS BDT trimer devices in ambient air

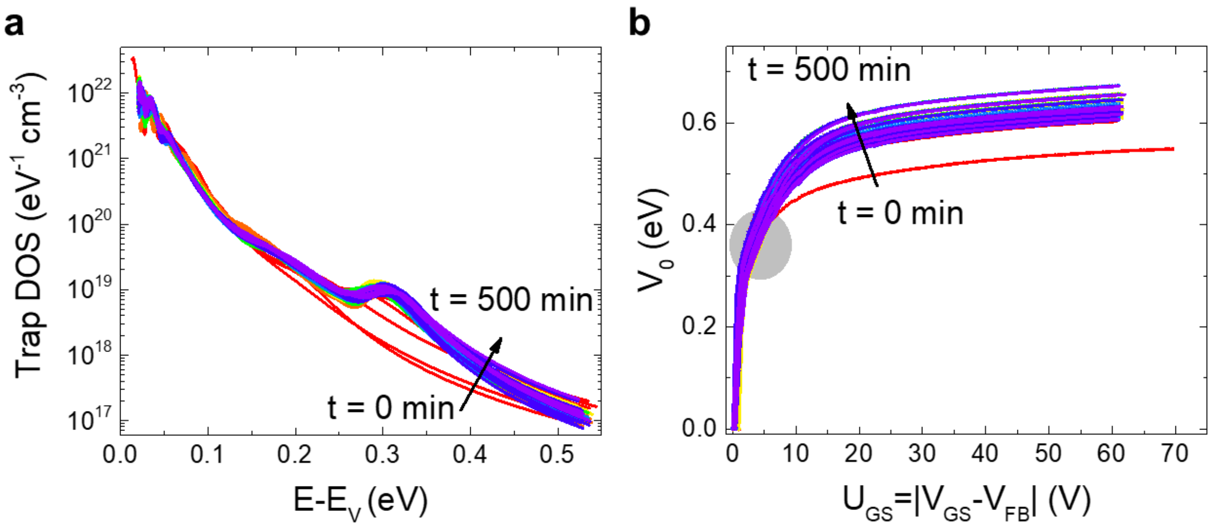


**Supplementary Figure 1 | Trap DOS analysis performed on an unencapsulated TnHS BDT trimer device operating in ambient air for 500 min. a,** DOS spectra evaluated during repeated transistor operation. **b,** The gate-voltage dependent interface potential function. The gray shaded area highlights a subtle change in the curvature of the plots giving rise to the peak in the corresponding DOS spectrum.

**Supplementary Table 1.** Model parameters used for the double exponential and Gaussian distributions employed to model the DOS spectra of a device operated in ambient air. The spectrum at t = 500 min was modelled using only a double exponential distribution and therefore the Gaussian parameters are not applicable.

| Model Parameter | t = 0 min | t = 500 min |
|---|---|---|
| $N_1$ (eV$^{-1}$ cm$^{-3}$) | $2.0 \times 10^{22}$ | $4.8 \times 10^{22}$ |
| $E_1$ (meV) | 25.0 | 17.5 |
| $N_2$ (eV$^{-1}$ cm$^{-3}$) | $5.2 \times 10^{19}$ | $5.0 \times 10^{20}$ |
| $E_2$ (meV) | 85.5 | 64.9 |
| $A$ (eV$^{-1}$ cm$^{-3}$) | N/A | $7.0 \times 10^{18}$ |
| $E_{peak}$ (eV) | N/A | 0.31 |
| $\sigma$ (meV) | N/A | 25.0 |

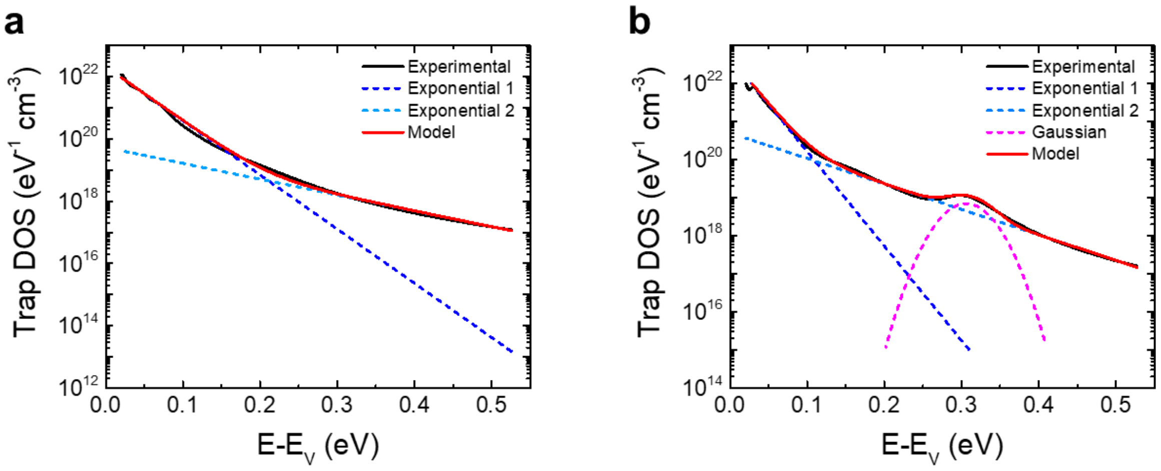


**Supplementary Figure 2 | Modeling of the trap DOS spectra of an unencapsulated TnHS BDT trimer device operating in ambient air.** Model fit (red solid lines) to experimental DOS spectrum (solid black lines) of a device at different times during operation: **a**, t = 0 min and **b**, after t = 500 min of operation. Broken lines represent the individual distribution functions employed for the modelling.

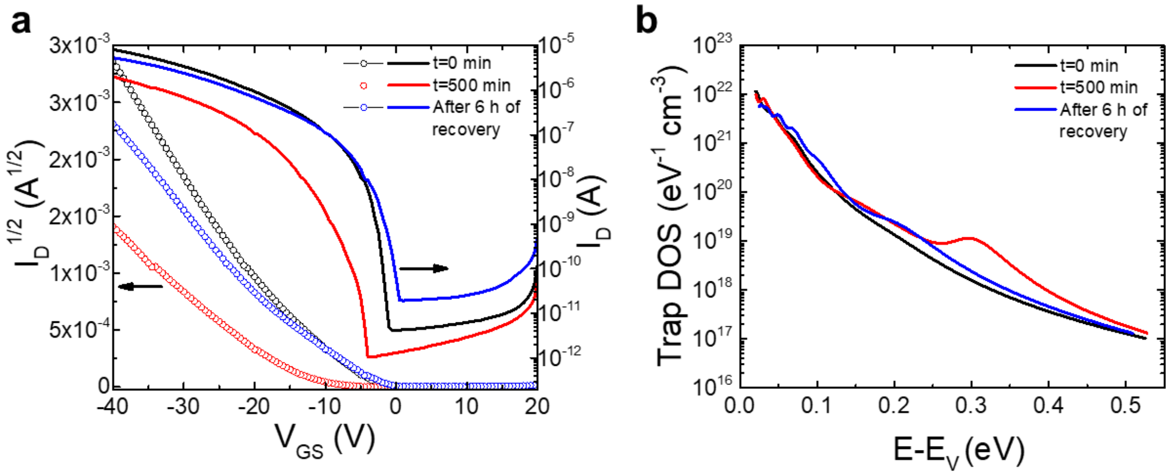


**Supplementary Figure 3 | Transistor characteristics of an unencapsulated TnHS BDT trimer device during repeated measurements and after recovery. a**, Saturation regime transfer characteristics ($V_{DS}$ =-60 V) of a device at t = 0 min (black), t = 500 min (red) of operation, and after 6 h of device recovery (blue). The left and right axes show the square root and the logarithm of $I_D$ respectively. **b**, Trap DOS spectra evaluated at t = 0 min (black), t = 500 min (red) of operation, and after 6 h of device recovery (blue).

## Supplementary Discussion 2: Operational stability tests on unencapsulated TnHS BDT trimer devices in vacuum

**Supplementary Table 2.** Model parameters used for the double exponential distributions employed to model the DOS spectra of a device operated in vacuum.

| Model Parameter | t = 0 min | t = 500 min |
|---|---|---|
| $N_1$ (eV$^{-1}$ cm$^{-3}$) | $1.5 \times 10^{22}$ | $7.4 \times 10^{20}$ |
| $E_1$ (meV) | 20.0 | 9.1 |
| $N_2$ (eV$^{-1}$ cm$^{-3}$) | $3.9 \times 10^{20}$ | $1.8 \times 10^{20}$ |
| $E_2$ (meV) | 53.4 | 47.5 |

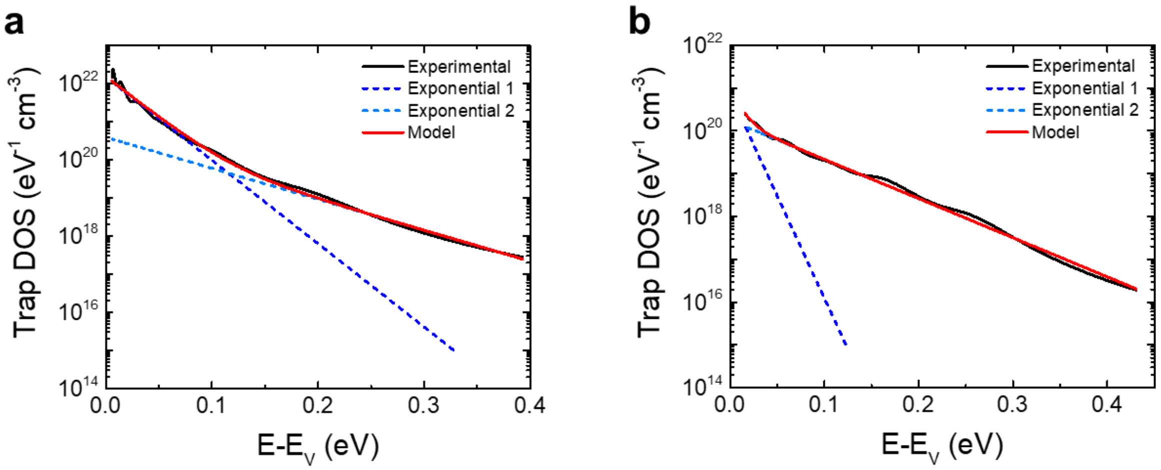


**Supplementary Figure 4 | Modeling of the trap DOS spectra of an unencapsulated device operating in vacuum.** Model fit (red solid lines) to experimental DOS spectrum (solid black lines) of a device at different times during operation: **a**, t = 0 min and **b**, after t = 500 min of operation. Broken lines represent the individual distribution functions employed for the modelling.

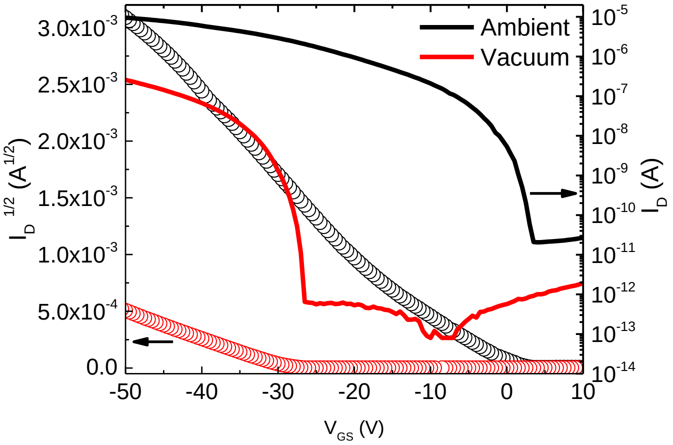


**Supplementary Figure 5 | Comparison of the current-voltage characteristics of an unencapsulated device measured under different environments.** Saturation regime transfer characteristics ($I_D$ vs $V_{GS}$ at $V_{DS}$=-60 V) under ambient air (black) and vacuum (red). The left and right axes show the square root and the logarithm of $I_D$ respectively.

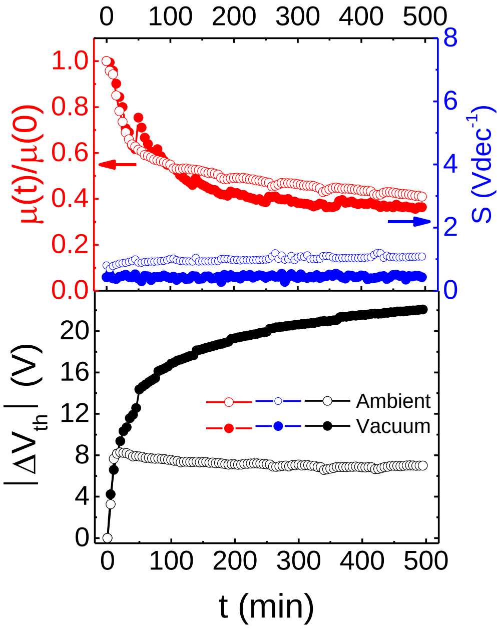


**Supplementary Figure 6 | Time evolution of device metrics of an unencapsulated TnHS BDT trimer device during repetitive transistor operation under different environments.** Open circles and solid circles represent measurements obtained in ambient air and vacuum, respectively. Time evolution of the mobility $\mu$ normalized to the value at t = 0 min (red), subthreshold slope $S$ (blue) and magnitude of threshold voltage shifts $|\Delta V_{th}|$ (black) are shown.

## Supplementary Discussion 3: Environmental stability tests on unencapsulated TnHS BDT trimer devices

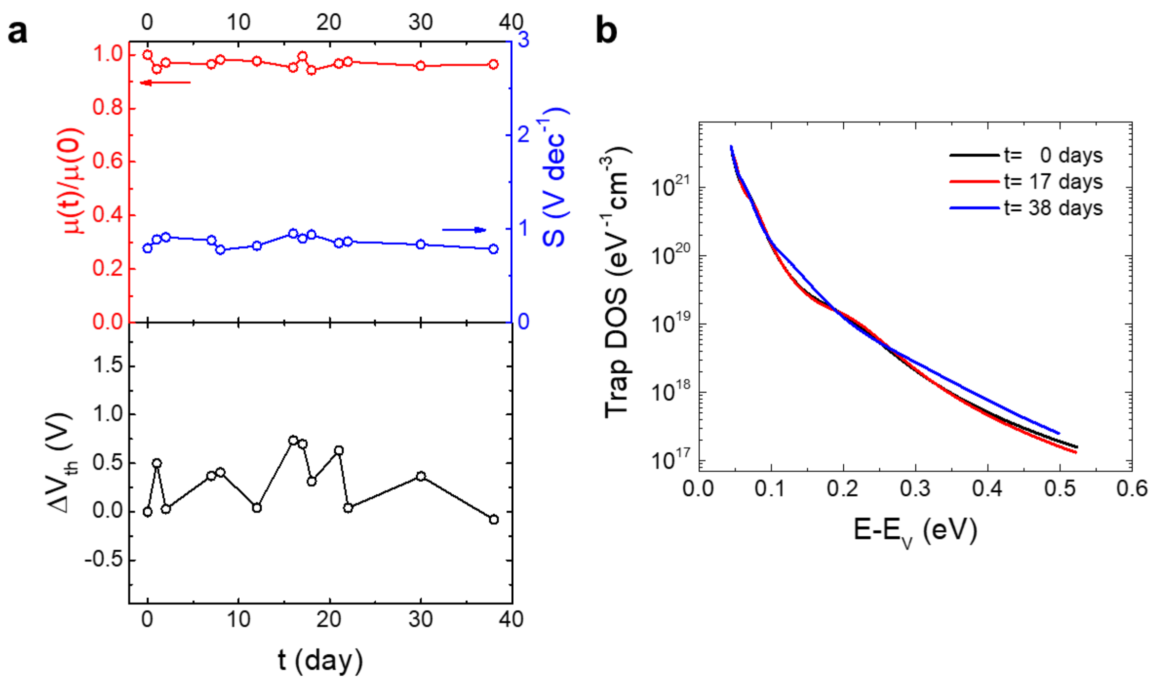


**Supplementary Figure 7 | Time evolution of device metrics and trap DOS spectrum of an unencapsulated TnHS BDT trimer device during environmental stability tests. a**, Time evolution of the mobility $\mu$ normalized to the value at t = 0 min (red), subthreshold slope $S$ (blue) and threshold voltage shifts $\Delta V_{\mathrm{th}}$ (black) are shown. **b**, Trap DOS spectra evaluated over the course of environmental stability tests performed for 38 days.

## Supplementary Discussion 4: Computational details

Density functional theory (DFT) calculations were carried out on isolated molecules and bulk crystal structures. Molecular calculations were performed at the ωB97XD/Def2SVP level of theory as implemented in Gaussian 16[3–5]. Bulk crystal calculations were carried out with Vienna *Ab-initio* Simulation Package[6–9], making use of the Perdew, Burke, and Ernzerhof exchange-correlation functional[10]. The electron-ion interactions were described with the projector augmented wave method[11]. The kinetic energy cutoff for the plane-wave basis set was set to 520 eV, and a Gaussian smearing with a width of 50 meV was employed. The D3 correction was employed with BJ-damping to describe the dispersion forces[12]. The convergence criterion of the total energy was set to $10^{-5}$ eV in the self-consistent field loop, and that of forces during relaxation was set to 0.01 eV/Å. Considering the large volume of the TnHS BDT trimer unit cell, Gamma centered 2 × 2 × 1 mesh was used to sample the Brillouin zone. Unit cells are also used for charged defects as the shortest translation vector is larger than 10 Å.

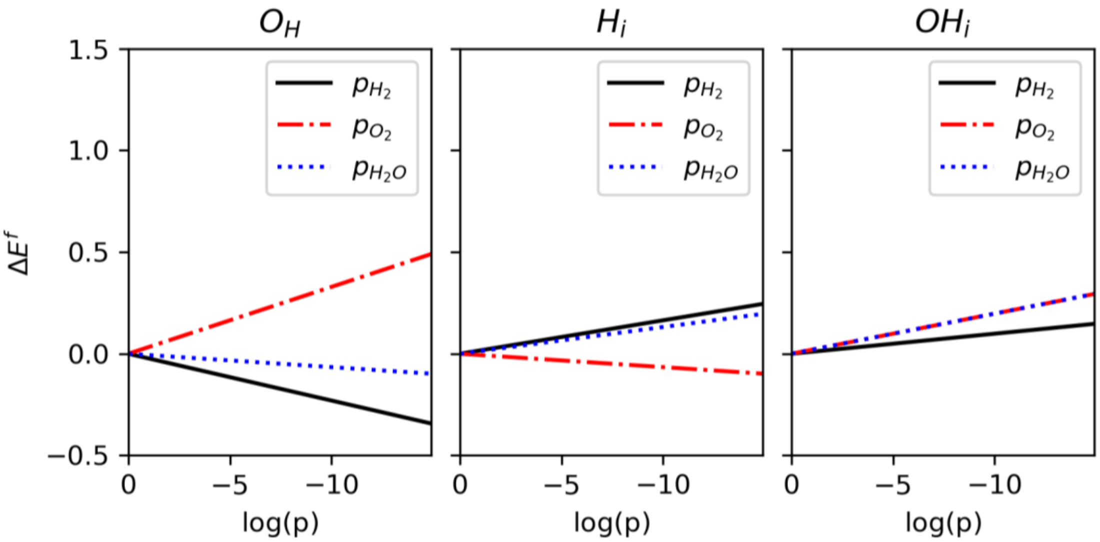


**Supplementary Figure 8 | Linear relationships between $\Delta E^f$ and $\log(P)$ for the three potential chemical defects of TnHS BDT trimer.**

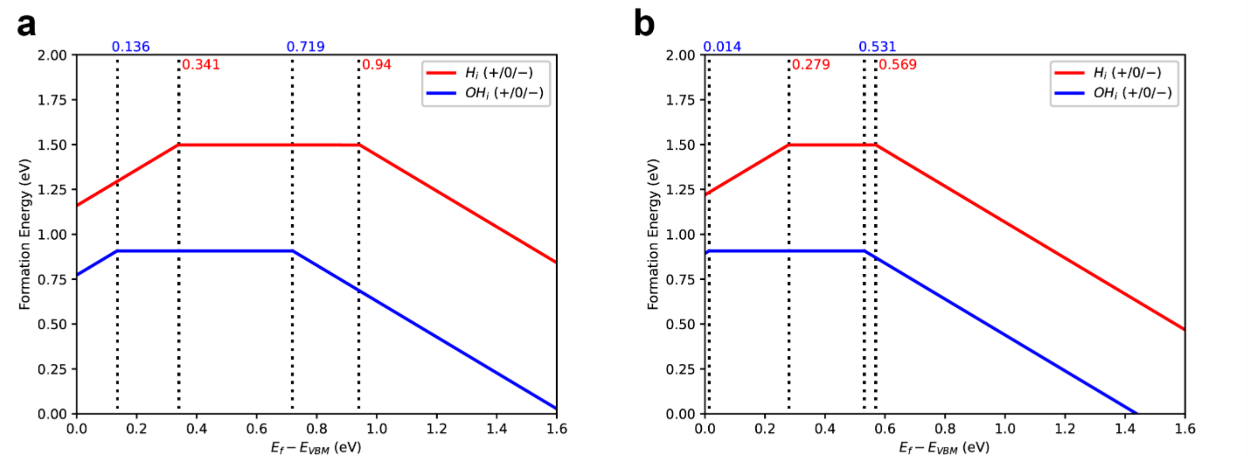


**Supplementary Figure 9 | Defect formation energies as a function of Fermi level relative to the valence band edge for $H_i$ and $OH_i$ defects in TnHS BDT trimer at $P_{O_2} = P_{H_2} = P_{H_2O} = 10^{-10}$ *Pa*. a**, Defect formation energies without correction and **b**, after applying the correction scheme (FNV) proposed by Freysoldt *et al.*[13,14], as implemented in PyCDT[15]. Transition levels are labeled with vertical dotted lines. Only monocation or monoanion states are considered.

## Supplementary Discussion 5: Operational stability tests on unencapsulated vs. encapsulated TnHS BDT trimer device in air

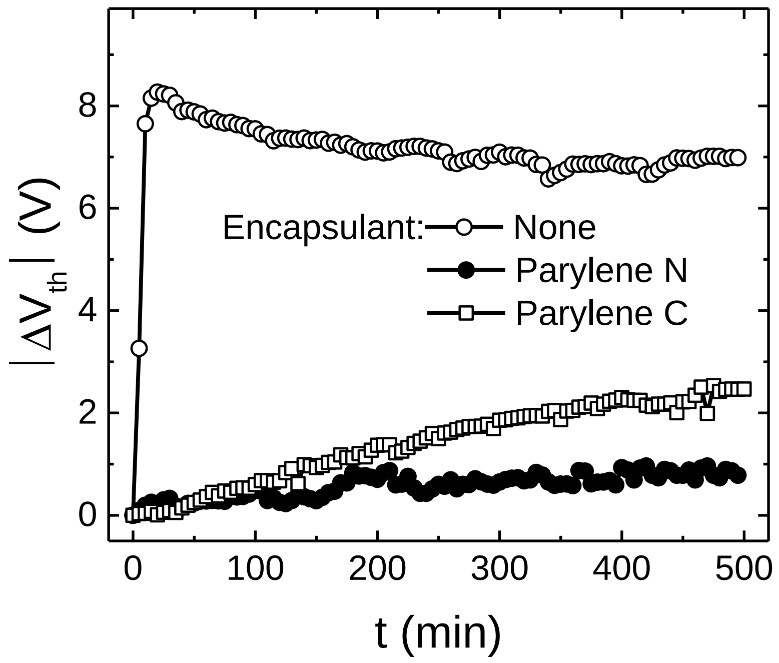


**Supplementary Figure 10 | Comparison of threshold voltage shifts of unencapsulated and encapsulated TnHS BDT trimer devices during repetitive transistor operation for 500 min in air.** An unencapsulated device (open circles) yielded a shift of $\Delta V_{th}$ = - 8 V while devices encapsulated with parylene N (solid circles) and parylene C (open squares) yielded shifts of $\Delta V_{th}$ = - 0.9 V and $\Delta V_{th}$ = - 2.5 V respectively.

## Supplementary Discussion 6: Hysteresis in current-voltage characteristics of TnHS BDT trimer devices

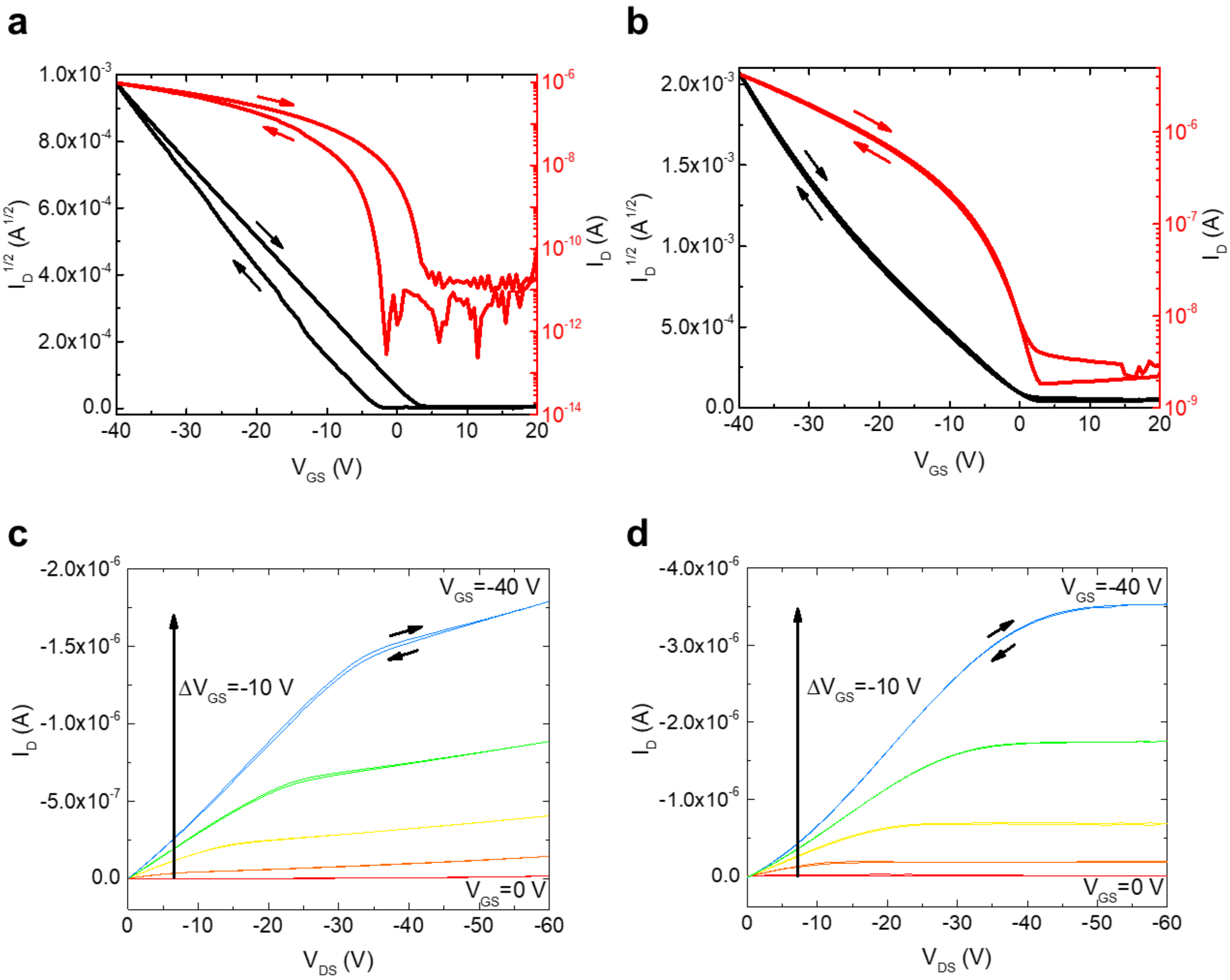


**Supplementary Figure 11 | Hysteresis in current-voltage characteristics of unencapsulated and parylene N-encapsulated TnHS BDT trimer devices**. **a**, Hysteresis in the transfer characteristics of an unencapsulated device in comparison to **b**, a device encapsulated with parylene N. **c**, Hysteresis in the transport characteristics of the unencapsulated device in comparison to **d**, the encapsulated device.

## Supplementary Discussion 7: Bias stress measurements on TnHS BDT trimer devices

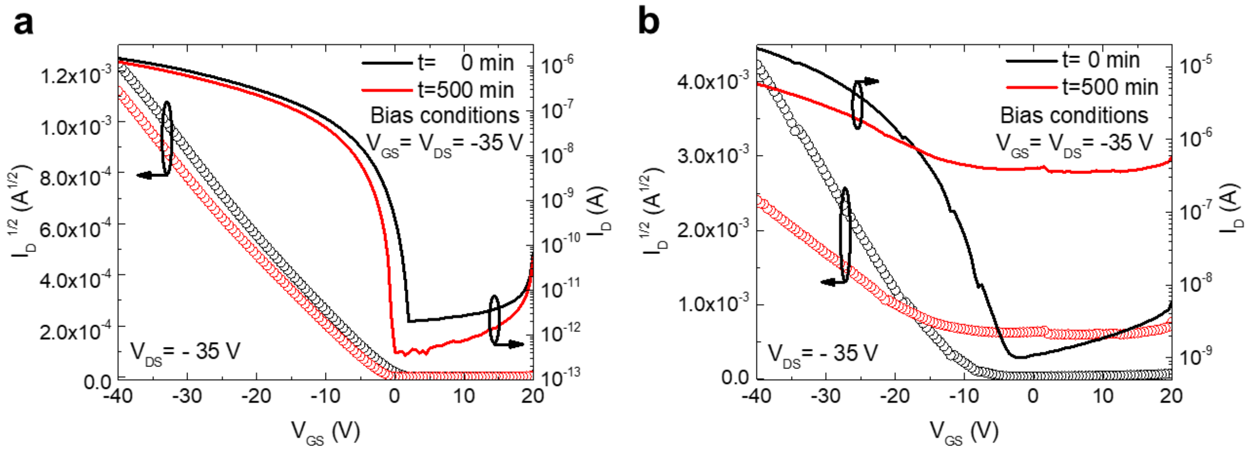


**Supplementary Figure 12 | Bias stress measurements on a TnHS BDT trimer device encapsulated with parylene C compared to an unencapsulated device. a,** Transfer characteristics of a device encapsulated with parylene C acquired at $V_{DS}$ = -35 V prior to stressing (black) and after 500 min of stressing (red) under the application of a continuous drain voltage and a dynamic gate bias pulsed at 10 s interval at $V_{DS} = V_{GS}$ = - 35 V. The threshold voltage shift is $\Delta V_{th}$ = - 1.3 V**. b,** Transfer characteristics of an unencapsulated device acquired at $V_{DS}$ = - 35 V prior to stressing (black) and after 500 min of stressing (red) under identical bias conditions. The threshold voltage shift is $\Delta V_{th}$ = + 4.9 V**.**

## Supplementary Discussion 8: Bilayer dielectric IDT-BT devices

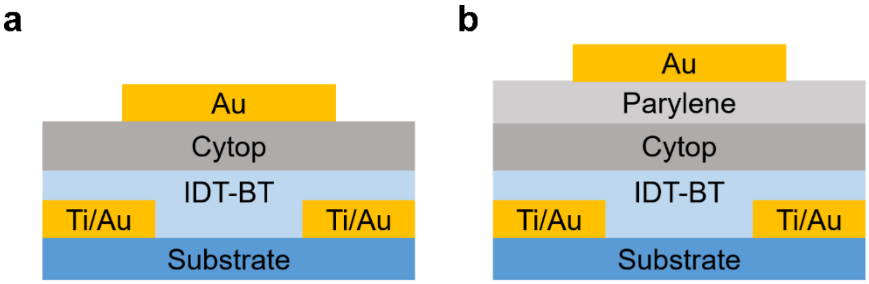


**Supplementary Figure 13 | IDT-BT device geometries fabricated and tested. a**, Bottom-contact, top-gate device with Cytop dielectric. **b,** Bottom-contact, top-gate device with bilayer dielectric consisting of Cytop and parylene.

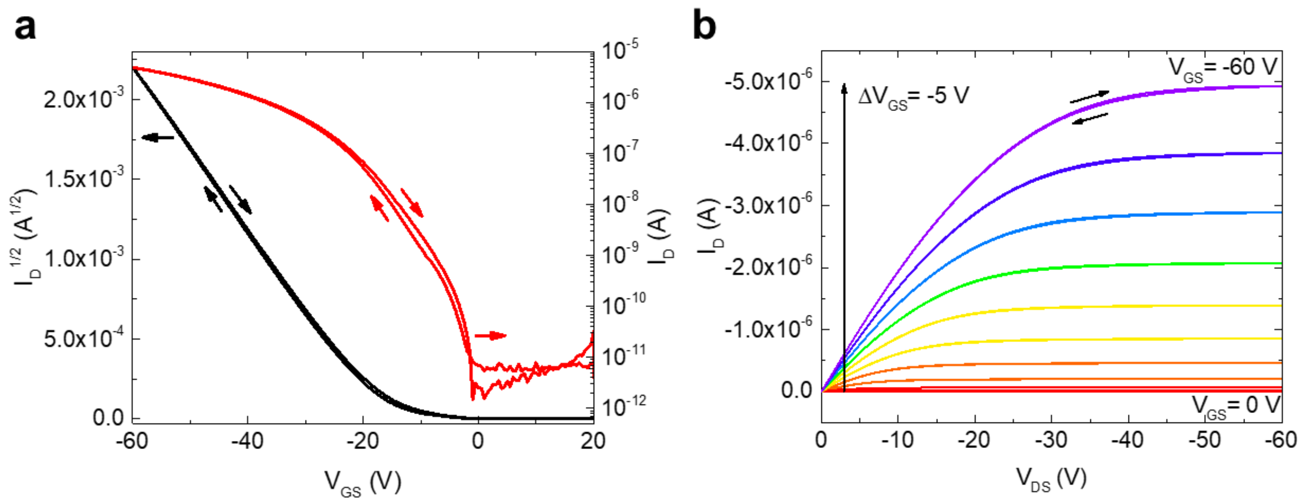


**Supplementary Figure 14 | Hysteresis in current-voltage characteristics of a bilayer dielectric IDT-BT device**.

**a**, Hysteresis in the transfer characteristics. **b**, Hysteresis in the output characteristics.

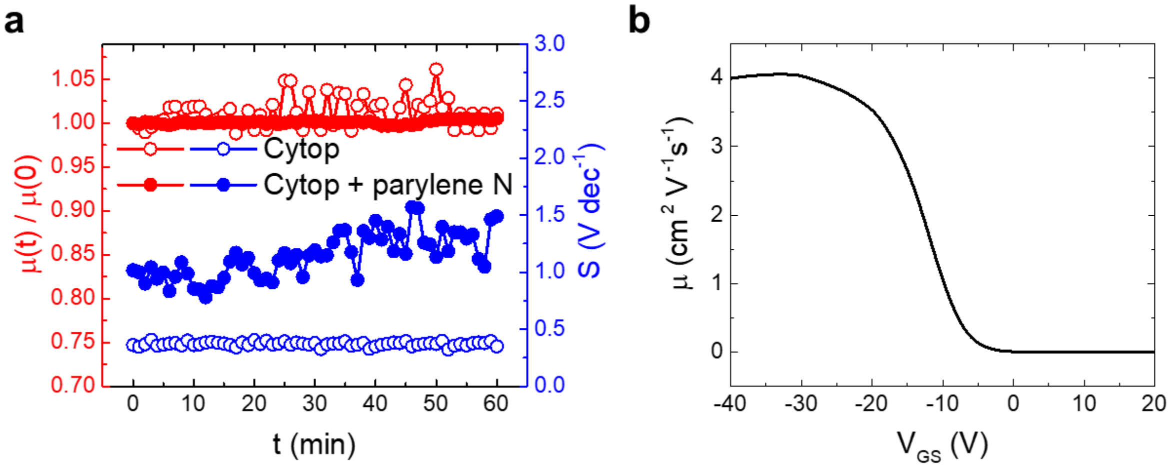


**Supplementary Figure 15 | Time evolution of device metrics and the dependency of mobility on gate-voltage in IDT-BT devices fabricated with different dielectrics. a**, Left and right axes show the change in normalized mobility and subthreshold slope respectively of a Cytop-only device (open circles) in comparison to a bilayer dielectric device (solid circles). **b**, Gate voltage dependence of the mobility of the bilayer dielectric device with $L$ = 100 µm, $W$ = 200 µm and an equivalent areal capacitance of $C_i$=1.26 nF cm$^{-2}$.

## Supplementary References


1. Massey, L. K. *Permeability Properties of Plastics and Elastomers, 2nd Ed.: A Guide to Packaging and Barrier Materials*. (Elsevier Science, 2003).
2. McKeen, L. W. *Permeability Properties of Plastics and Elastomers: Fourth Edition*. *Permeability Properties of Plastics and Elastomers: Fourth Edition* (Elsevier Science, 2016).
3. Chai, J.-D. & Head-Gordon, M. Long-range corrected hybrid density functionals with damped atom–atom dispersion corrections. *Phys. Chem. Chem. Phys.* **10**, 6615 (2008).
4. Dunning, T. H. Gaussian basis sets for use in correlated molecular calculations. I. The atoms boron through neon and hydrogen. *J. Chem. Phys.* **90**, 1007–1023 (1989).
5. Frisch, M. J. *et al.* G16_C01. Gaussian 16, Revision C.01, Gaussian, Inc., Wallin (2016).
6. Kresse, G. & Hafner, J. Ab initio molecular dynamics for liquid metals. *Phys. Rev. B* **47**, 558–561 (1993).
7. Kresse, G. & Joubert, D. From ultrasoft pseudopotentials to the projector augmented-wave method. *Phys. Rev. B* **59**, 1758–1775 (1999).
8. Kresse, G. & Furthmüller, J. Efficient iterative schemes for ab initio total-energy calculations using a plane-wave basis set. *Phys. Rev. B* **54**, 11169–11186 (1996).
9. Kresse, G. & Furthmüller, J. Efficiency of ab-initio total energy calculations for metals and semiconductors using a plane-wave basis set. *Comput. Mater. Sci.* **6**, 15–50 (1996).
10. Perdew, J. P. *et al.* Erratum: Atoms, molecules, solids, and surfaces: Applications of the generalized gradient approximation for exchange and correlation. *Phys. Rev. B* **48**, 4978–4978 (1993).
11. Blöchl, P. E. Projector augmented-wave method. *Phys. Rev. B* **50**, 17953–17979 (1994).
12. Grimme, S., Ehrlich, S. & Goerigk, L. Effect of the damping function in dispersion corrected density functional theory. *J. Comput. Chem.* **32**, 1456–1465 (2011).
13. Freysoldt, C., Neugebauer, J. & Van de Walle, C. G. Fully Ab Initio Finite-Size Corrections for

Charged-Defect Supercell Calculations. *Phys. Rev. Lett.* **102**, 016402 (2009).

14. Freysoldt, C., Neugebauer, J. & Van de Walle, C. G. Electrostatic interactions between charged defects in supercells. *Phys. status solidi* **248**, 1067–1076 (2011).

15. Broberg, D. *et al.* PyCDT: A Python toolkit for modeling point defects in semiconductors and insulators. *Comput. Phys. Commun.* **226**, 165–179 (2018).
